# Assessing the Impact of Comprehensive Genomic Profiling on Therapeutic Selection for Advanced Solid Tumors in Portugal

**DOI:** 10.3390/curroncol33020066

**Published:** 2026-01-23

**Authors:** Nuno Tavares, Pedro Simões, Raquel Lopes-Brás, Teresa R. Pacheco, Sara Damaso, Andre Mansinho, Leonor Abreu Ribeiro, Gonçalo Nogueira-Costa, Catarina Abreu, Tiago Barroso, Nuno Bonito, Rita Figueiró, Bogdana Darmits, Sara Loureiro Melo, Tania Rodrigues, Helena Guedes, Edgar Pratas, Diogo Alpuim Costa, Frederico Ferreira Filipe, Daniela Macedo, Ana Cavaco, Marina Pavanello, Luis Costa

**Affiliations:** 1 Medical Oncology Department, Unidade Local de Saúde São João, 4200-319 Porto, Portugal; nuno.tavares@ulssjoao.min-saude.pt; 2 Medical Oncology Department, Unidade Local de Saúde Loures-Odivelas, 2674-514 Loures, Portugal; pedro.simoes@ulslod.min-saude.pt; 3 Medical Oncology Department, Unidade Local de Saúde Santa Maria, 1649-035 Lisboa, Portugalteresa.pacheco@ulssm.min-saude.pt (T.R.P.); sarafdamaso@gmail.com (S.D.); andre.mansinho@startlisbon.com (A.M.); leonor.ribeiro@ulssm.min-saude.pt (L.A.R.); goncalo.costa@ulssm.min-saude.pt (G.N.-C.); catarina.abreu@ulssm.min-saude.pt (C.A.); tiago.barroso@ulssm.min-saude.pt (T.B.); marina.pavanello@ulssm.min-saude.pt (M.P.); 4 Gulbenkian Institute for Molecular Medicine, 1649-028 Lisboa, Portugal; acmcavaco@gmail.com; 5 Faculty of Medicine, University of Lisbon, Av. Prof. Egas Moniz, 1649-028 Lisboa, Portugal; 6 Medical Oncology Department, Instituto Português de Oncologia de Coimbra Francisco Gentil, 3000-075 Coimbra, Portugal; 3544@ipocoimbra.min-saude.pt (N.B.); 4214@ipocoimbra.min-saude.pt (R.F.); 4215@ipocoimbra.min-saude.pt (B.D.); 4213@ipocoimbra.min-saude.pt (S.L.M.); 7 Medical Oncology Department, Hospital da Luz, 1500-650 Lisboa, Portugal; trodrigues@hospitaldaluz.pt; 8 Medical Oncology Department, Unidade Local de Saúde de Gaia e Espinho, 4434-502 Vila Nova de Gaia, Portugal; helena.guedes@ulsge.min-saude.pt; 9 Medical Oncology Department, Unidade Local de Saúde de Coimbra, 3004-561 Coimbra, Portugal; 10557@ulscoimbra.min-saude.pt; 10 Hematology and Oncology Department, CUF Oncologia, 1998-018 Lisboa, Portugal; diogo.costa@cuf.pt; 11 Oncology Functional Unit, Hospital de Cascais, 2755-009 Alcabideche, Portugal; frederico.ferreira.filipe@hospitaldecascais.pt; 12 Medical Oncology Department, Hospital Lusíadas, 1500-458 Lisboa, Portugal; daniela.gouveia.macedo@lusiadas.pt

**Keywords:** precision oncology, comprehensive genomic profiling, solid tumors

## Abstract

**Background:** Comprehensive genomic profiling (CGP) is a tool used in precision oncology to identify genomic alterations and match them with targeted therapies across several tumor types. However, real-world data on its clinical utility and impact remains limited. The FRONTAL study (Foundation Medicine Real wOrld evideNce in porTugAL) is a multicenter academic initiative that established a national registry of Portuguese patients with solid tumors who underwent CGP with FoundationOne CDx, Liquid CDx or FoundationOne Heme assays. **Methods:** Eligible patients had advanced solid tumors not suitable for curative treatment at the time of recruitment. Prior CGP testing was permitted if taken within 12 months before study initiation. Genomic profiling data were extracted from FoundationOne Medicine reports, and clinical information was extracted from medical records. Actionable alterations were defined as those associated with approved treatments or with clinical evidence of benefit in other cancers, per NCCN guidelines. Variant interpretation was also reviewed according to ESMO Scale for Clinical Actionability of Molecular Targets (ESCAT) guidelines. The primary outcome was disease control at 16 weeks, defined by the absence of progression. **Results:** The study included 205 patients between 2020 and 2025 across 10 sites, with colorectal (40, 19.5%), sarcomas (28, 13.7%), and other gastrointestinal tumors (22, 10.7%) being the most common pathologies. Actionable alterations were identified in 104 cases (50.7%). Genomic findings guided therapy decisions in 50 patients (24.4%), of whom 30 achieved disease control at 16 weeks (14.6%). **Conclusions:** The FRONTAL study highlighted the clinical relevance of CGP in advanced solid tumors. Over half of the patients had actionable alterations, a quarter had therapy changes based on CGP results, and improved disease outcome was observed in approximately 15% of the cohort.

## Simple Summary

Real-world evidence data on the clinical impact of comprehensive genomic profiling (CGP) remains limited. The FRONTAL study, a multicenter national registry in Portugal, evaluated the use of CGP in patients with advanced solid tumors who underwent testing with FoundationOne CDx, Liquid CDx, or FoundationOne Heme. Among 205 patients enrolled between 2020 and 2025, actionable genomic alterations were identified in half of the cases, and CGP results influenced treatment decisions in about one-quarter of patients. Importantly, approximately 15% of the total cohort achieved disease control at 16 weeks following CGP-guided therapy. Overall, this study provided real-world evidence supporting the clinical relevance of CGP in informing treatment strategies for advanced solid tumors in Portugal.

## 1. Introduction

The paradigm of precision oncology has fundamentally transformed cancer therapeutics, emphasizing the integration of molecular characterization into clinical decision-making [1,2]. Comprehensive genomic profiling (CGP) enables the simultaneous detection of a wide spectrum of genomic alterations across multiple cancer-related genes, providing a broader molecular landscape than conventional targeted gene testing, which is inherently limited by its focus on predefined alterations.

Among the most established diagnostic tools, the FoundationOne^®^ platforms—CDx [3], Liquid CDx [4], and Heme [5]—offer analytically and clinically validated CGP assays for solid tumors, circulating tumor DNA, and sarcomas, respectively. These platforms facilitate the identification of actionable genomic alterations and support the implementation of targeted therapies within routine clinical practice.

Currently, the European Society for Medical Oncology (ESMO) recommends the use of tumor CGP testing for several advanced solid tumors initially, including non-squamous non-small cell lung cancer, prostate, colorectal, cholangiocarcinoma, and ovarian cancers [6]. Updated guidance has expanded these recommendations to include advanced breast cancer and selected rare tumors such as gastrointestinal stromal and thyroid tumors, sarcoma, and cancers of unknown primary [6]. In addition, CGP is advised to identify tumor-agnostic alterations in metastatic cancers when matched targeted therapies are available [6].

Several studies have been investigating evidence to support the use of CGP in the management of oncology patients. Studies such as SHIVA [7], MOSCATO-01 [8], and ProfiLER [9] have demonstrated that 40%, 49%, and 52% of patients, respectively, had at least one actionable molecular alteration with the potential to guide targeted therapy. However, these studies did not demonstrate significant improvements in clinical outcomes among patients treated with CGP-matched therapies. Importantly, many of these prospective trials of multigene sequencing shared the limitation of excluding patients whose tumors harbor genomic alterations for which approved targeted therapies already exist. In addition, basket trials have also been conducted to evaluate the activity of targeted therapies across multiple tumor types that share a common molecular alteration. The NCI-MATCH trial demonstrated that large-scale, multi-site molecular screening is feasible, enabling testing of investigational therapies for a range of molecular targets, and successfully met its signal-seeking goal, with 25.9% of substudies showing a positive result [10]. The TAPUR trial (NCT02693535), which is currently recruiting, aims to evaluate the antitumor activity of commercially available targeted agents in patients with advanced cancers harboring genomic alterations known to be drug targets [11]. Within the European context, the Danish ProTarget trial (NCT04341181) is currently evaluating the antitumor activity and safety of 13 EMA-approved targeted agents used beyond their approved indications in patients with advanced cancers harboring specific actionable genomic alterations [12]. Similarly, the Dutch DRUP trial (NCT 02925234) employs a comparable basket-trial design, further underscoring the need to assess the clinical utility of CGP in routine oncology practice [13].

Given the increasing incorporation of CGP into oncology workflows, generating real-world evidence (RWE) is also essential to evaluate its clinical utility, assess patient outcomes, and inform evidence-based precision oncology [14]. Recent studies have reported favorable outcomes in patients receiving targeted therapies guided by CGP testing. In 2023, a comprehensive review of more than 6000 publications identified 31 studies that evaluated progression-free and overall survival in patients with advanced tumors treated according to CGP findings [15]. This review concluded that CGP-guided therapy can positively impact survival across tumor types [15]. More recently, in 2025, a RWE meta-analysis including 144 studies demonstrated that nearly 60% of patients harbored at least one actionable alteration, with approximately 15% ultimately receiving targeted therapy [14]. About one-fourth of patients treated with CGP-matched therapy achieved an objective response, and CGP-guided treatment was associated with prolonged progression-free survival (pooled hazard ratio [HR] = 0.63; 95% CI = 0.56–0.70; 18 studies) as well as overall survival (pooled HR = 0.60; 95% CI = 0.51–0.70; 21 studies) [14].

In this study, we aimed to establish a national registry to better understand health outcomes resulting from the integration of CGP in clinical practice in Portugal. We characterized the clinical and demographic profile of Portuguese patients with malignant solid tumors that underwent CGP analysis by FoundationOne testing to describe their genomic landscape (including both actionable and non-actionable variants), assess the relevance of CGP results for subsequent therapeutic decision-making, and evaluate patient outcomes under therapies selected based on CGP results.

## 2. Materials and Methods

### 2.1. Study Cohort and Workflow

The Foundation Medicine Real wOrld evideNce in PorTugAL (FRONTAL) study is a multicenter observational study conducted at the national level between 2020 and 2025. Patients with confirmed diagnosis of solid malignant neoplasms unsuitable for curative treatment at the time of recruitment and who had undergone CGP were considered for inclusion. For patients with CGP results already available at inclusion, testing must have been performed within 12 months prior to study initiation. Informed consent from the patient or their legal representative was mandatory for living patients, in accordance with local regulatory requirements. For deceased patients, informed consent was waived in accordance with applicable ethical guidelines. All centers had the study protocol approved by their respective Ethics Committee. CGP testing were requested on a case-by-case basis according to the treating physician’s judgement, as testing is not routinely implemented or reimbursed in most Portuguese hospitals and was therefore limited by financial constraints.

At the time of inclusion, baseline data were collected for each patient. This included demographic information, details of the primary tumor diagnosis (e.g., date of diagnosis, tumor type, grade, and stage), curative treatments received prior to study entry, and palliative treatments until the CGP testing. After baseline data collection, follow-up real-world clinical data were retrieved from the medical records. The initial follow-up focused on the medical visit during which CGP results were reviewed. It was documented whether a targeted treatment was proposed based on the genomic findings and if a discussion had taken place in a Molecular Tumor Board. Subsequent follow-up data was retrieved every four months to assess treatment response until death, loss to follow-up, withdrawal of consent, or discontinuation from the registry. In addition, quality of life was assessed during follow-up consultations with medical oncologists (when possible) using the EORTC QLQ-C30, a validated 30-item instrument designed to evaluate quality of life in all cancer patients.

### 2.2. Comprehensive Genomic Profiling

All samples were tested using FoundationOne^®^ CDx [3], FoundationOne Liquid Biopsy [4], and FoundationOne Heme [5] (Foundation Medicine, Inc., Cambridge, MA, USA). Testing, data processing, and variant calling were performed in accordance with the manufacturer’s instructions and previously published validation studies [3,4,5]. Sequence data were analyzed using a purpose-built, analytically validated bioinformatics pipeline developed by Foundation Medicine to detect all supported classes of genomic alterations (substitutions, insertion and deletions (indels), splicing), including structural variants, copy number alterations (CNAs) such as amplifications and homozygous deletions, and selected genomic rearrangements such as gene fusions. In addition, the assays report genomic signatures, including microsatellite instability (MSI) and tumor mutational burden (TMB).

FoundationOne CDx detected substitutions, indels, and CNAs in 324 genes. It also detected MSI, TMB, as well as loss of heterozygosity (LOH) score for ovarian cancer samples. DNA was isolated from formalin-fixed, paraffin-embedded (FFPE) tumor tissue.

FoundationOne Liquid Biopsy detected substitutions and indels in 311 genes, CNAs in 310 genes, and rearrangements in 324 genes, in accordance with the manufacturer’s instructions. It also detected blood tumor mutational burden (bTMB), MSI, and tumor fraction. This test used circulating cell-free DNA (cfDNA) isolated from plasma collected from anticoagulated whole blood of cancer patients. Both FoundationOne CDx and FoundationOne Liquid CDx are FDA-approved companion diagnostics for all solid tumors, with indications covering 15 to 35 targeted therapies.

FoundationOne Heme was used to test sarcoma patients in the FRONTAL study. It detected substitutions, indels, CNAs, and rearrangements in 406 genes. It also analyzed selected introns of 31 genes using FFPE tumor tissue. In addition, RNA sequencing of 265 genes was used to detect gene fusions, which are common drivers of sarcomas and other hematologic malignancies.

### 2.3. Variant Interpretation

FoundationOne reports summarized publicly available evidence matching specific molecular alterations, or their absence, to drugs with potential clinical benefit, including investigational therapies [16]. Each report was curated based on international clinical guidelines (e.g., National Comprehensive Cancer Network (NCCN), American Society of Clinical Oncology (ASCO), ESMO, World Health Organization (WHO)), peer-reviewed literature, conference resources, and genomic databases (e.g., PubMed, ClinVar, and COSMIC) [16].

In addition to the data provided in the FoundationOne report, variant interpretation was reviewed as part of this study using the ESMO Scale for Clinical Actionability of Molecular Targets (ESCAT) [17]. Molecular alterations were classified into six tiers based on clinical evidence and relevance to patient management, ranging from tier I (ready for routine clinical use) to tier X (no evidence of actionability). Tier II included targets with likely clinical benefit but requiring additional data, while tier III referred to targets with benefit demonstrated in other tumor types or for analogous molecular alterations. Additionally, tier IV was supported by preclinical evidence, and tier V encompassed targets with a rationale for co-targeting strategies rather than standalone therapeutic relevance. Within the aim of this study, variants classified with tiers I to III were considered actionable variants.

### 2.4. Outcome and Clinical Data Assessment

Therapeutic response was retrospectively assessed using electronic health records and classified according to standard clinical practice and physician decision-making. The primary outcome was disease control at 16 weeks, defined by the absence of progression.

## 3. Results

### 3.1. Clinical Characteristics of Patients

**Figure 1:**
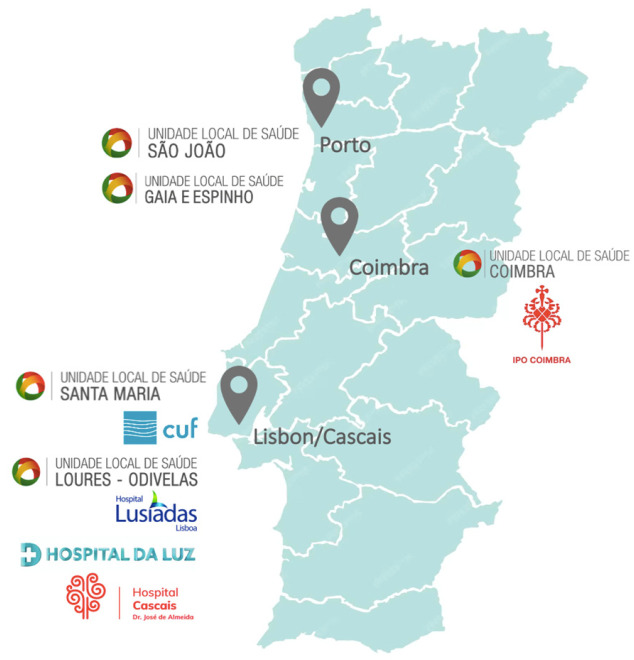
Regional distribution of the participating sites in the study.

A total of 205 patients were enrolled in the study between 2020 and 2025 from 10 participating sites distributed across different regions of Portugal (Figure 1).

The distribution of recruited patients according to the type of healthcare institution can be found in Table 1.

Data on biological sex, race, patient status (alive vs. deceased), family history of cancer, tobacco use, and alcohol consumption were collected for all patients and are shown in Table 2.

Biological sex distribution was balanced, with 110 males (53.7%) and 95 females (46.3%). Most patients were identified as White (n = 168, 82.0%), while Black or African Descent individuals accounted for 4 cases (2.0%), and Asian individuals for 1 case (0.5%). Race was not recorded for 32 patients (15.6%). Regarding patient status, 161 individuals were deceased (78.5%), and 44 were alive (21.5%) at final data review. A family history of cancer was reported in 87 cases (42.4%), absent in 77 (37.6%), and unknown in 41 (20.0%). Regarding tobacco use history, 120 patients (58.5%) had never used tobacco, while 66 (32.2%) were previous or current users. Tobacco use status was unknown for 19 patients (9.3%). Alcohol consumption was absent in 174 cases (84.9%), present in 13 (6.3%), and undocumented in 18 (8.8%).

**Figure 2:**
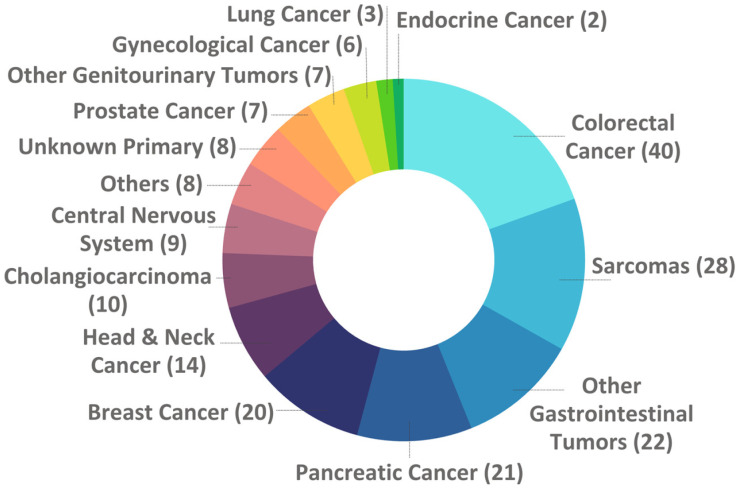
FRONTAL cohort according to tumor types.

Figure 2 illustrates the distribution of cancer types among patients included in the study cohort.

The most prevalent malignancy was colorectal cancer (n = 40, 19.5%), followed by sarcomas (n = 28, 13.7%), other gastrointestinal tumors (n = 22, 10.7%), and pancreatic cancer (n = 21, 10.2%) (Figure 2). Breast cancer accounted for 20 cases (9.8%), while head and neck cancers comprised 14 cases (6.8%). Cholangiocarcinoma was identified in 10 patients (4.9%), and central nervous system tumors in nine (4.4%). Cancers of unknown primary were recorded in eight cases (3.9%). Prostate cancer and other genitourinary tumors each represented seven cases (3.4%), gynecological cancers were identified in six cases (2.9%), lung cancer in three cases (1.5%), and endocrine cancer in two cases (1.0%). Finally, a combined category labeled as Others were classified in eight cases (3.9%), which included the following tumor types: ameloblastoma, porocarcinoma of the skin, undifferentiated carcinoma of the mediastinum, squamous cell carcinoma of the skin, melanoma of the ciliary body, melanoma not otherwise specified, basal cell carcinoma of the skin, synchronous carcinomas of the cecum (ileocecal region) and sigmoid colon.

All patients presented with metastatic or unresectable disease at the time of the CGP test. However, at initial diagnosis, 39.5% of the cohort had stage I–III disease, and 49.3% had stage IV disease (Table 3). The disease stage at diagnosis was unknown or not reported in the electronic health records for 11.2% of patients.

At the time of diagnosis, 58% of patients in the cohort received treatment with curative intent, whereas 42% started a palliative treatment line as their initial therapeutic approach (Table 3). The median number of palliative lines of treatment until the CGP was three, within a range of zero and 11. Seventeen patients in the cohort performed CGP without having received any prior line of palliative treatment. Among these, nine had sarcomas, three presented with head and neck tumors, and one case each corresponded to breast cancer, cholangiocarcinoma, pancreatic cancer, endometrial carcinoma, and an undifferentiated carcinoma of the mediastinum.

Information regarding the clinical purpose of CGP was also collected throughout the study. Table 4 presents this data stratified by aggregated tumor categories.

For the tumor aggregation, colorectal, pancreatic, cholangiocarcinoma, and other gastrointestinal tumors were combined under the Gastrointestinal category (n = 93). Breast and gynecologic malignancies were combined into a single Breast & Gynecological group (n = 26), while prostate tumors were grouped with other urological neoplasms under Urological (n = 14). Head and neck cancers were combined with lung tumors as the Head & Neck/Respiratory category (n = 17). The remaining tumor types, including sarcomas (n = 28), central nervous system tumors (n = 9), cancers of unknown primary (n = 8), and a heterogeneous group labeled as Others (n = 10), were retained as previously defined. As shown in Table 4, most tests (84.4%) were performed in cases with few available treatment options, followed by rare tumors that accounted for 10.7% of tests. Testing for cancers of unknown primary origin comprised 3.9% of cases, and only 1.0% of tests were performed for advanced tumors diagnosed at a young age.

### 3.2. Molecular Testing

Regarding the CGP specimen type, most of the testing was derived from primary tissue tumors (n = 84, 41.0%), followed by peripheral blood samples (n = 62, 30.2%), and metastatic tissue samples (n = 53, 25.9%). Local recurrences accounted for seven specimens (3.4%). One patient had both primary and metastatic samples tested. Although no major clinically significant differences were observed between the two tests, the primary tumor sample revealed a greater number of gene alterations, resulting in a slightly higher tumor mutational burden. Interestingly, the additional alterations consisted exclusively of amplifications. These were identified in the following genes: *CDK6*, *HGF*, *MYC*, *CDK8*, *CUL4A*, *DIS3*, *FGF14*, *FLT3*, and *IRS2*. Single-nucleotide variants and/or frameshifts in *KRAS*, *APC*, and *TP53*, as well as a wild-type profile in *NRAS*, were identified in both samples.

**Figure 3:**
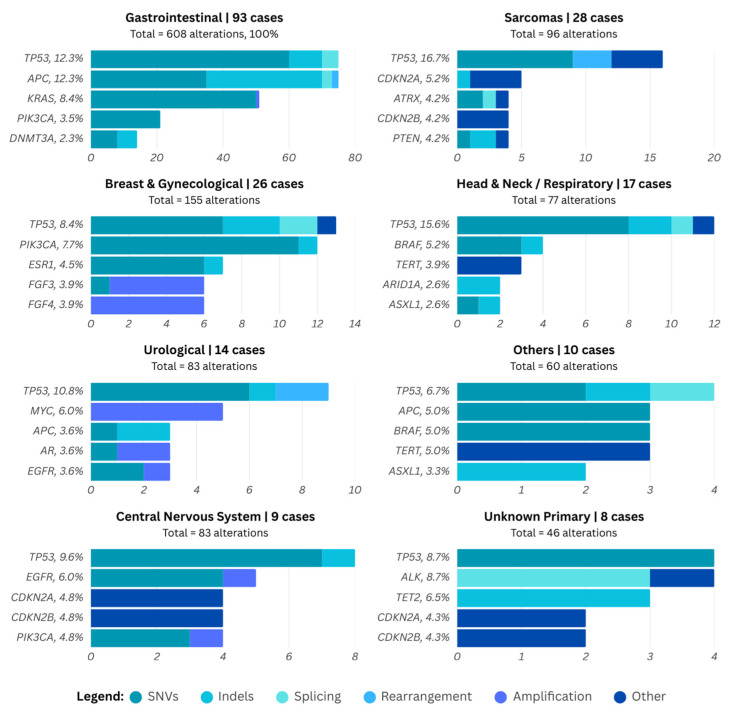
Most frequently altered genes stratified by aggregated tumor type and alteration classification.

In total, 117 patients (56.8%) underwent testing with the FoundationOne CDx assay, 62 (30.1%) with the FoundationOne Liquid Biopsy, and 27 (13.1%) with the FoundationOne Heme assay. Figure 3 shows the distribution of the most commonly altered genes across aggregated tumor types. For each gene, the frequency of alteration relative to the total number of alterations is shown, with stacked bars indicating the number and type of genomic alterations within each tumor group.

As shown in Figure 3, in gastrointestinal tumors, the most frequent alterations were *APC* and *TP53*, each detected in 12.3% of cases, followed by *KRAS* (8.4%), *PIK3CA* (3.5%), and *DNMT3A* (2.3%). Among sarcomas, *TP53* was also the predominant alteration (16.7%), with additional findings in *CDKN2A* (5.2%), *ATRX* (4.2%), *CDKN2B* (4.2%), and *PTEN* (4.2%). In breast and gynecological tumors, *TP53* (8.4%) and *PIK3CA* (7.7%) were the most frequently altered genes, followed by *ESR1* (4.5%), *FGF3* and *FGF4* (each 3.9%). Head and neck and respiratory tumors showed alterations in *TP53* (15.6%) and *BRAF* (5.2%), with lower frequencies observed for *TERT (3.9%)*, *ARID1A*, and *ASXL1* (each 2.6%).

In addition, in urological tumors, the most frequently altered gene was *TP53* (10.8%), followed by *MYC* (6.0%), with *APC*, *AR*, and *EGFR* each observed in 3.6% of cases. Among tumors classified as Others, alterations were most commonly found in *TP53* (6.7%), *APC*, *BRAF*, and *TERT* (each 5.0%), and with *ASXL1* present in 3.3% of cases. In central nervous system tumors, *TP53* was again the most frequently altered gene (9.6%), followed by *EGFR* (6.0%), *CDKN2A, CDKN2B*, and *PIK3CA* (each 4.8%). Finally, for cancers of unknown primary, the most frequent alterations were in *TP53* and *ALK* (8.7% each), followed by *TET2* (6.5%), and *CDKN2A*/*B* (each 4.3%).

Regarding molecular signatures, tumor mutational burden (TMB) and microsatellite instability (MSI) status were assessed in all cases, while homologous recombination deficiency (HRD) was additionally evaluated in gynecological tumors. A total of 28 patients (13.7%) were identified with either TMB > 10 mutations/Mb, MSI-high status, or HRD-associated loss of heterozygosity (HRD-LOH). Among these, three patients presented with both TMB-high and MSI-high profiles, 24 had TMB-high only, and one case was positive for HRD-LOH.

### 3.3. Outcomes

Overall, among the 205 patients profiled, 104 (50.7%) presented with at least one actionable molecular alteration, while the remaining 101 (49.3%) had no actionable molecular alterations identified. Of the 104 patients with actionable molecular alterations, 56 received targeted treatment recommendations due to the CGP finding, corresponding to 27.3% of the cohort. Importantly, five patients died before they could begin the recommended therapy and one patient refused the proposed targeted treatment. Consequently, a total of 50 patients ultimately received targeted therapy aligned with their CGP findings (24.4%). Of the 50 patients who received therapy guided by CGP, 30 maintained disease control at 16 weeks–representing 60.0% of those treated with CGP-matched therapy and 14.6% of all patients included in the study. Table 5 highlights the patients with disease control and their characteristics.

Among patients with controlled disease, the most frequently observed molecular alterations were MSI and/or TMB high, accounting for 20.0% of all alterations. These were followed by *BRAF* mutations (16.7%), mutations in *BRCA1/2* and *BRIP1* (10.0%), *PIK3CA* mutations (10.0%), and *ALK* alterations (6.7%).

In addition, we compared disease outcomes according to each molecular alteration identified in these patients who received CGP-matched therapy (Table 6).

Among the genomic alterations evaluated in patients who received CGP-matched therapies, tumors with *BRAF* mutations, *PIK3CA* mutations, and *ALK* alterations demonstrated higher rates of disease control (Table 6). In addition, alterations such as MSI and/or high TMB, *BRCA1/2* and *BRIP1* mutations, and *ERBB2* amplification were evenly distributed between controlled and uncontrolled disease cases. Poorer outcomes were observed in patients with *ATM* and *NF1* mutations, where disease control was less frequent. The remaining 13 cases showed a diverse range of genomic alterations. Of these, nine cases with controlled disease included *EGFR* amplification, *ESR1* mutation, *FBXW7* mutation, *FGFR2* rearrangement, *HRAS* mutation, *KIT* mutation, *NF1* large deletion, *NTRK3* fusion, and *VEGFA* amplification. The remaining four cases with uncontrolled disease were characterized by *GNA11*, *KRAS*, *PDGFRA*, and *PTCH1* mutations.

The average number of prior palliative treatment lines was also assessed in patients with controlled disease compared to those without disease control. Both groups showed a mean value of 2.1 prior treatment lines, suggesting that the number of previous treatment lines did not influence outcomes in the FRONTAL cohort.

In addition, we investigated whether patients without any prior palliative treatment at the time of CGP had actionable alterations and subsequently received targeted therapy. As shown in Table 3, 17 patients had received no palliative lines before CGP testing. Among these, eight patients were found to have actionable alterations, and three received targeted treatment. One case was a *BRAF*-mutated head and neck tumor treated with dabrafenib and trametinib, achieving disease control for 84 weeks. The other two cases were *PIK3CA*-mutated tumors treated with everolimus: a gynecological case with disease control for 19 weeks, and a sarcoma case without disease control (progressed within 13 weeks).

### 3.4. Molecular Tumor Boards

For this cohort, Molecular Tumor Boards (MTB) were not consistently established across all centers during the study period. Therefore, of the 205 patients included in this study, 48 cases were discussed at MTB, accounting for 23.4% of the cases. Of those 48 cases, 25 (12.2%) had an actionable alteration according to the CGP, and 13 (6.3%) were offered a targeted therapy. Two patients deceased before receiving the targeted therapy.

To further explore these findings, outcomes were compared between cases discussed in MTB and those that were not. Among the 48 cases reviewed in MTB, 25 (52.1%) had actionable alterations, and 11 (22.9%) received targeted treatment. Of these, seven cases achieved disease control, while four did not. Similarly, of the 157 cases not discussed in MTB, 79 (50.3%) harbored at least one actionable alteration, and 39 (24.8%) received targeted therapy. Of these 39 patients, 23 achieved disease control. Although the total number of cases differed substantially between the two groups, the relative frequencies were consistent between the groups and with the overall cohort: approximately half of the cases carried actionable alterations, and about one-quarter received targeted treatment based on CGP results. The same trend was observed in treatment outcomes, with both MTB-discussed and non-discussed cases demonstrating a higher proportion of disease control at 16 weeks, consistent with the overall cohort findings described previously.

### 3.5. Quality of Life Evaluation

One of the aims of the FRONTAL study was to assess quality of life in patients who received targeted therapy based on CGP compared with those who did not. However, the collection of EORTC QLQ-C30 questionnaires was challenging in routine hospital practice. Quality-of-life data were available for only 38 patients (18.5% of the cohort) and importantly, these assessments were not consistently obtained at each follow-up visit. In addition, only four of the patients with reported quality-of-life data had received targeted therapy, which did not allow comparative analysis as part of this study.

## 4. Discussion

Among the 205 patients enrolled in the FRONTAL study between 2020 and 2025 across 10 sites, actionable alterations were identified in 104 patients (50.7%), and these genomic findings guided therapy decisions in 50 patients (24.4%). Among patients receiving targeted therapy based on CGP, 30 (14.6%) achieved disease control at 16 weeks.

The FRONTAL study demonstrated results that were relatively consistent with previously published data, reinforcing the reliability of its findings. A recent retrospective analysis of 79 breast and gynecological tumors reported that next-generation sequencing informed treatment recommendations in 63.3% of cases, whereas the corresponding rate in the FRONTAL study was 50.7% [18]. Furthermore, 29.1% of patients in the referenced research received molecularly matched therapy, compared to 24.4% in our cohort [18]. Additionally, a recent meta-analysis of 144 real-world evidence studies reported that 59.8% of CGP-tested patients were identified with actionable genomic alterations, while 15.6% ultimately received molecularly matched therapies [14]. Importantly, it is known that the proportion of patients receiving targeted therapy following CGP varies across published studies due to several factors [19,20,21]. Not receiving a certain molecularly matched treatment despite its CGP finding could be due to physician or patient preference for alternative therapeutic options, lack of reimbursement, limited drug availability, and disease progression leading to poor performance status or death prior to treatment initiation [19,20,21]. These barriers underscore the complexity of translating genomic findings into clinical benefit and highlight the need to integrate CGP results into therapeutic decision-making in a timely manner.

The results of this study were also consistent with the existing literature regarding the most frequently detected genes overall and across different tumor types. Although the included pathologies were heterogeneous and, for the purposes of this study, broadly grouped into larger categories, the findings aligned with what is known and expected. Overall, the most commonly altered gene was *TP53*, which is well established as the most frequently mutated gene across tumor types [22]. When stratified by tumor type, high frequency of *TP53* mutations was also observed as expected, including in colorectal cancer [23], sarcomas [24], as well as in breast [25,26], gynecological [27,28,29], head and neck [30], prostate [31], central nervous system tumors such as glioblastomas [32], and cancers of unknown primary origin [33]. In gastrointestinal tumors (mostly colorectal and pancreatic cancers), which represented the largest cancer cohort in the FRONTAL study, the other most frequently mutated genes were *APC* [34] and *KRAS* [35], in line with what is expected for these tumor types. Additionally, a high frequency of *PIK3CA* mutations was observed in breast cancers [26], further supporting consistency with published data.

In the Portuguese context, the use of CGP testing to support therapeutic decision-making is endorsed by the national Medical Council. In 2023, a national guideline was published in alignment with the recommendations of the ESMO Translational Research Group, providing guidance for medical oncologists in defining treatment strategies [36]. In our national cohort, nearly half of the patients were found to have at least one targetable molecular alteration. However, it is worth mentioning that the most common molecular findings were expected given the tumor types included, and many actionable variants could also be detected with smaller targeted molecular profiling panels already available in Portugal. The added value of broader and more expensive CGP panels remains uncertain and should be further evaluated, including whether shorter in-house panels may offer comparable clinical utility in guiding therapy according to actionable alterations.

Another important area of interest is the use of CGP in clinical trials. According to ESMO guidelines, CGP testing should be used to facilitate patient enrolment and to support drug development [6]. In a recently published Belgian cohort, out of 756 patients with successful CGP analysis, 422 (55.8%) were recommended to participate in biomarker-driven clinical trials [37]. This contrasts with the Portuguese reality, where, despite the fact that the number of clinical trials has been increasing in recent years, as documented by the Portugal Clinical Studies database (https://portugalclinicalstudies.com/index.php/en/portugal-numbers, accessed on 4 December 2025) [38], trial opportunities are scarce and regional asymmetries remain a challenge, particularly regarding early-phase basket trials, which could substantially expand access for patients with actionable genomic alterations to receive targeted therapies within clinical trial settings. These data reinforce the importance of investigating how CGP results may enhance patient recruitment into Portuguese clinical trials.

The best timing in the course of cancer disease to perform comprehensive genomic testing is still debatable. In our cohort, only 8.3% of patients were tested in the first line setting. Recent data showed that performing a CGP in patients with previously untreated advanced solid tumors can detect actionable mutations in 100% of patients, druggable alterations in 63.4% and molecular-based recommended therapies can be offered in molecular tumor boards in 61.0% [39]. We can question whether performing CGP early in the advanced course of the disease can provide relevant information in a timely manner to consider the most appropriate therapeutic sequencing. Moreover, this topic can be further complicated by the choice of tumor sample for genomic profiling, namely whether CGP should be performed on the primary tumor, on an initial metastatic lesion, or on metastatic tissue obtained after failure of standard-of-care therapies. Still, data regarding the clinical benefit obtained with these targeted therapies will be critical to assess the relevance of early CGP use.

In addition, our data showed that in 84.4% of the cases, CGP testing was requested when few treatment options were available. Given this high proportion, it might be worth considering that for tumors with limited therapeutic options and rapid growth patterns, CGP testing likely needs to be performed at earlier disease stages to provide better opportunities for patients. In our study, for instance, five patients underwent CGP testing and were identified as eligible for matched targeted therapy; however, they died before the CGP-matched treatment could be initiated. In summary, our data further reinforced the need for institutional protocols that ensure reimbursement of genomic testing within the National Health Service and support adherence to national guidelines, allowing testing to be performed at the appropriate time and in the appropriate patients. As previously mentioned, in Portugal, these national guidelines correspond to the 2023 recommendations issued by the Portuguese Medical Council [36], which aligned with ESMO guidance for CGP use in advanced solid tumors [6]. In addition, this study also highlighted the importance of expanding precision oncology programs to broaden access to genomic panels and strengthening collaboration between oncology centers to enable timely referral to early-phase clinical trials.

Lastly, although evaluating quality of life was one of the objectives of the FRONTAL study, data were successfully collected in only a small subset of patients, corresponding to 18.5% of the entire cohort. This limitation highlighted the need for a more structured implementation of the protocol. In future studies, it may be more feasible and efficient to restrict quality-of-life assessments to the subset of patients who undergo therapeutic modification based on genomic profiling, which in our study represented 24.4% of the cohort, although some of these cases were recruited retrospectively. For patients recruited prospectively, this targeted approach could more effectively capture whether CGP-driven decisions translate into measurable improvements in patients’ quality of life, an area where evidence remains critically needed.

## 5. Conclusions

The FRONTAL study was the first real-world evidence study in Portugal to establish a national registry of patients with malignant solid tumors who underwent CGP analysis, aiming to describe their clinical characteristics and tumor genomic profiles, as well as to assess the relevance of CGP findings for subsequent therapeutic decision-making. These results may support clinicians in determining whether comprehensive genomic profiling is the most appropriate diagnostic approach and in identifying the optimal timing for its implementation within the oncology care pathway.

## Figures and Tables

**Table 1:** Number of patients recruited by healthcare institutions.

Health System Category	Number of Patients
University Hospital ^(a)^	128
General Hospital ^(b)^	32
Private Hospital ^(c)^	27
Oncology Institute ^(d)^	18
Total	205

^(a)^ sites: Unidade Local de Saúde (ULS) São João, ULS Santa Maria, and ULS Coimbra; ^(b)^ sites: ULS Loures-Odivelas, ULS Gaia e Espinho, and Hospital de Cascais; ^(c)^ sites: Hospital Luz Lisboa, CUF, and Lusíadas; ^(d)^ site: IPO Coimbra.

**Table 2:** Patient characteristics in the FRONTAL study.

	N	Percentage (%)
Total	205	100
	Biological Sex	
Male	110	53.7
Female	95	46.3
	Race	
White	168	82.0
Black or African Descent	4	2.0
Asian	1	0.5
Unknown/Not reported	32	15.6
	Patient Status	
Deceased	161	78.5
Alive	44	21.5
	Family History of Cancer	
Yes	87	42.4
No	77	37.6
Unknown	41	20.0
	Tobacco Use	
Never	120	58.5
Previous or Current	66	32.2
Unknown	19	9.3
	Alcohol Consumption	
Never	174	84.9
Previous or Current	13	6.3
Unknown	18	8.8

**Table 3:** Disease and treatment characteristics of the FRONTAL cohort.

	**Tumor Stage at Diagnosis**	**%**
I–III	81	39.5
IV	101	49.3
Unknown/Not reported	23	11.2
	Initial therapeutic intention	%
Curative Intent	119	58.0
Palliative Intent	86	42.0
	Number of Palliative Lines of Treatment until CGP	%
0	17	8.3
1	52	25.4
2	52	25.4
3	42	20.5
>3	42	20.5
	3 (median)	0–11 (range)

**Table 4:** Purpose of the comprehensive genomic test by tumor type.

Tumor Type	Purpose of the Test				Total
	Few Treatment Options	Rare Tumor	Unknown Primary	Advanced Tumor at Young Age	
Gastrointestinal	86	6	NA	1	93
Sarcomas	27	1	NA	NA	28
Breast & Gynecological	25	NA	NA	1	26
Head & Neck/Respiratory	11	6	NA	NA	17
Urological	11	3	NA	NA	14
Others	5	5	NA	NA	10
Central Nervous System	8	1	NA	NA	9
Unknown Primary	NA	NA	8	NA	8
Total	173 (84.4%)	22 (10.7%)	8 (3.9%)	2 (1.0%)	205

NA: not available.

**Table 5:** List of patients with disease controlled in the FRONTAL study.

FRONTAL ID	Cancer Type	Actionable Alteration	CGP-Targeted Treatment	Disease Control (in Weeks)
220	Head and Neck Cancer	*NTRK3* fusion	Entrectinib	149
219	Head and Neck Cancer	*BRAF* non-V600 mutation	Trametinib	126
124	Head and Neck Cancer	*FBXW7* mutation	Everolimus	95
1	Head and Neck Cancer	*BRAF* V600E mutation	Dabrafenib and Trametinib	84
149	Head and Neck Cancer	*ERBB2* amplification	Trastuzumab	80
138	Others	*ATM* mutation	Niraparib	52
108	Gynecological Cancer	TMB high	Pembrolizumab	45
159	Head and Neck Cancer	*BRAF* V600E mutation	Sorafenib & Dabrafenib-Trametinib	45
52	Others	*BRAF* V600E mutation	Dabrafenib & Trametinib	45
123	Pancreatic Cancer	*BRAF* V600E mutation	Dabrafenib & Trametinib	37
179	Colorectal Cancer	*BRCA1/2* & *BRIP1* mutations	Talazoparib	36
153	Colorectal Cancer	*PIK3CA* mutation	Alpelisib	35
11	Other Gastrointestinal Tumors	MSI and TMB high	Pembrolizumab	35
213	Cholangiocarcinoma	*HRAS* mutation	Trametinib	35
207	Endocrine Cancer	TMB high	Pembrolizumab	33
210	Colorectal Cancer	*NF1* large deletion	Trametinib	30
90	Cholangiocarcinoma	TMB high	Pembrolizumab	30
161	Unknown Primary Tumors	*ALK* rearrangement	Entrectinib	26
76	Others	*EGFR* amplification	Anti-EGFR	25
189	Cholangiocarcinoma	*FGFR2* rearrangement	Pemigatinib	25
120	Cholangiocarcinoma	TMB high	Durvalumab plus Gemcitabine and Cisplatin	24
151	Colorectal Cancer	*PIK3CA* mutation	Alpelisib	22
217	Other Gastrointestinal Tumors	*BRCA2* mutation	Olaparib	20
5	Head and Neck Cancer	TMB high	Nivolumab and Ipilimumab	20
111	Breast Cancer	*ESR1* mutation	Abemaciclib and Fulvestrant	19
172	Pancreatic Cancer	*BRCA2* mutation	Olaparib	19
112	Gynecological Cancer	*PIK3CA* mutation	Everolimus and Letrozole	19
182	Colorectal Cancer	*VEGFA* amplification	Sorafenib	18
70	Sarcomas	*ALK* fusion	Alectinib	17
145	Other Gastrointestinal Tumors	*KIT* mutation	Sorafenib	17

**Table 6:** Disease outcome comparison by molecular alterations in patients who received CGP-matched therapy.

Molecular Alteration	Controlled Disease	%	Not Controlled Disease	%	Total
MSI and/or TMB High	6	50.0	6	50.0	12
*BRAF* mutations	5	83.3	1	16.7	6
*BRCA1/2* & *BRIP1* mutations	3	50.0	3	50.0	6
*PIK3CA* mutations	3	75.0	1	25.0	4
*ATM* mutations	1	33.3	2	66.7	3
*ALK* alterations	2	100.0	0	0.0	2
*ERBB2* amplification	1	50.0	1	50.0	2
*NF1* mutations	0	0.0	2	100.0	2
*EGFR* amplification	1	100.0	0	0.0	1
*ESR1* mutation	1	100.0	0	0.0	1
*FBXW7* mutation	1	100.0	0	0.0	1
*FGFR2* rearrangement	1	100.0	0	0.0	1
*HRAS* mutation	1	100.0	0	0.0	1
*KIT* mutation	1	100.0	0	0.0	1
*NF1* large deletion	1	100.0	0	0.0	1
*NTRK3* fusion	1	100.0	0	0.0	1
*VEGFA* amplification	1	100.0	0	0.0	1
*GNA11* mutation	0	0.0	1	100.0	1
*KRAS* mutation	0	0.0	1	100.0	1
*PDGFRA* mutation	0	0.0	1	100.0	1
*PTCH1* mutation	0	0.0	1	100.0	1
Total	30	60.0	20	40.0	50

## Data Availability

The data presented in this study are available on request from the corresponding author due to the sensitive clinical and genomic data and the need to protect participant privacy.

## Abbreviations

The following abbreviations are used in this manuscript:

ASCO	American Society of Clinical Oncology
bTMB	Blood tumor mutational burden
cfDNA	Circulating cell-free DNA
CGP	Comprehensive genomic profiling
CNA	Copy Number Alterations
ESCAT	ESMO Scale for Clinical Actionability of Molecular Targets
ESMO	European Society for Medical Oncology
FFPE	Formalin-Fixed Paraffin-Embedded
FRONTAL	Foundation Medicine Real wOrld evideNce in PorTugAL
HR	Hazard Ratio
HRD	Homologous Recombination Deficiency
Indels	Insertion and deletion alterations
LOH	Loss of Heterozygosity
MSI	Microsatellite Instability
MTB	Molecular Tumor Boards
NCCN	National Comprehensive Cancer Network
RWE	Real-World Evidence
TMB	Tumor Mutational Burden
